# Late-life psychiatric factors and life satisfaction are associated with cognitive errors: evidence from an experimental module of a large-scale survey in India

**DOI:** 10.1038/s41598-024-76180-9

**Published:** 2024-10-29

**Authors:** C. V. Irshad, P. Padma Sri Lekha, E. P. Abdul Azeez, T. Muhammed

**Affiliations:** 1 School of Social Sciences and Languages, Vellore Institute of Technology, Vellore, Tamil Nadu 632014 India; 2 Department of Human Development and Family Studies, Pennsylvania State University, University Park, PA 16802 USA

**Keywords:** Cognitive errors, Psychiatric factors, Depression, Life satisfaction, Cognitive impairment, Older adults, Psychology, Health care

## Abstract

Older adults are at risk of committing cognitive and decision-making errors due to the decline in cognitive functions. However, the understanding of the determining factors of cognitive errors among ageing adults is limited. In this study, we explored the role of various psychiatric factors, life satisfaction, and other socioeconomic, health and behavioural risk factors of committing cognitive errors among middle-aged and older adults in India. The study utilized the data from the experimental module of the Longitudinal Ageing Study in India (LASI) Wave-1 (2017–2018) with a sample of 12,754 adults aged 45 years and above. The cognitive error was measured using logical fallacies committed in the activity-based experiments. The study employed descriptive, bivariate statistics and multivariable logistic regression models to identify the factors associated with cognitive errors among the study participants. Depression (aOR = 1.28, 99%, CI: 1.16–1.41), life satisfaction (aOR = 0.99, 99%, CI: 0.98–1.00), and cognitive impairment (aOR = 1.13, 90% CI: 1.00–1.30) were significantly associated with higher odds of committing cognitive errors among the middle-aged and older adults. Also, ageing adults with low educational levels, functional limitations, sleep disturbances, smoking history, living in rural areas and belonging to scheduled tribes had a higher probability of committing cognitive errors. However, involvement in physical activity, reading habits and social interactions reduced the odds of cognitive errors among this sample. Mental health and well-being indicators, including depression, life satisfaction, cognitive impairment, and other health and behavioural health factors, determine cognitive errors among ageing adults. Programs and policies should be initiated to address these factors, reduce cognitive errors, and ensure active ageing.

## Introduction

Cognitive abilities facilitate individuals in navigating complex decision-making, problem-solving, and reasoning in everyday life^1^. Decision-making is a dynamic skill^2^, and it is influenced by various factors, such as past life experiences^3,4^, cognitive biases^5,6^, individuals’ personalities^7,8^, and beliefs about the importance of the problem they encounter ^9^. A study indicated that traits related to behavioural and cognitive self-regulatory functions and cognitive flexibility are related to an increased tendency to be involved in rational thoughts^7^. The decision-making process is guided by internal and external factors like perceptions and environment, and cognitive bias plays a crucial role^5^. Decision-making is an important faculty of cognition and is associated with various life outcomes^10^, including cognitive errors. Cognitive error is a volatile concept, and scholars conceptualize it differently.

Cognitive errors are common in everyday life, as no system is completely foolproof, and decision-making as a process of cognition also involves mental shortcuts (heuristics) to save resources and avoid cognitive overload in a world flooded with information. Even though heuristics helps in quicker decision-making and problem-solving, they are bound to errors and biased judgments^11^. A heuristic procedure is a collection of steps or rules that may or may not lead to an ideal optimal solution, and the steps are usually based on one’s intuitions, good ideas and hunches, problem characteristics, and reasoning processes^12^. Heuristics are effective during time constraints, lack or overload of information when no optimal solution is evident, and when the problem looks familiar^13^. A few commonly employed heuristics during uncertainty are representativeness, availability of instances or scenarios, and adjustment from an anchor. These are highly economical and generally effective but may lead to predictable and systematic errors^14^.

Interestingly, there is a significant association between ageing and cognitive capacities as decision-making or the trajectory of decision-making changes with age^15,16^. The decline in cognitive capacities like memory^17^, perceptual processing^18^, and executive functions^19^ while ageing may impact the process of decision-making and lead to related errors^2^. The literature points out the difference in affective-motivational factors during ageing. The major focus of decision-making during a young age is gain, but changes to prevent loss in later life^15,20^. Also, a study found that loneliness and cognitive distortion negatively impact life satisfaction and the interconnectedness between these factors^21^. Psychiatric conditions such as depression^22,23^ and neurological issues such as cognitive impairments^24–26^ affect decision-making and cognitive errors. Interestingly, a sense of purpose acted as a psychological resource, leading to fewer cognitive failures among adults, even countering the depressive effects^27^.

In addition, multiple psychosocial factors influence the process of decision-making and play a role in cognitive errors among adults, including interpersonal relationships^28^, stress^29^, health status^30^, economic well-being^31^, risky lifestyle behaviours^32^ and physical activities^33^. Further, a study among older adults in Japan suggested that participation in sports club activities is related to the articulation of decisions about life-prolonging treatments^34^, indicating better use of cognitive resources. As the concept of neural compensation suggests, being active and keeping the neural network active while ageing helps better physical and psychological functioning^35^. It is important to note that educational attainment adds to the cognitive reserve and brain maintenance, ultimately improving and facilitating better cognitive functions later in life^36^.

It has been widely established that older adults are at higher risk of committing decision-making errors due to reduced cognitive functions, and despite these odds, they must make decisions in their everyday lives^2^. Strikingly, a study showed that decision-making errors increased mortality risk by 20% among older adults^37^. Also, cognitive errors are associated with severe anxiety and depressive symptoms in older adults^38^, and subjective cognitive failures were associated with anxiety, depression, and anger^39^. In addition, cognitive biases were persistent across all economic groups regarding financial decisions^40^, indicating its relevance to be addressed. Further, the dynamic process of decision-making and cognitive errors was influenced by culture^41^. In the Indian context, cognitive bias is investigated from the business and investors’ perspectives^42,43^ with a minuscule focus on the general population, leading to a significant gap in this subject matter.

**Fig. 1:**
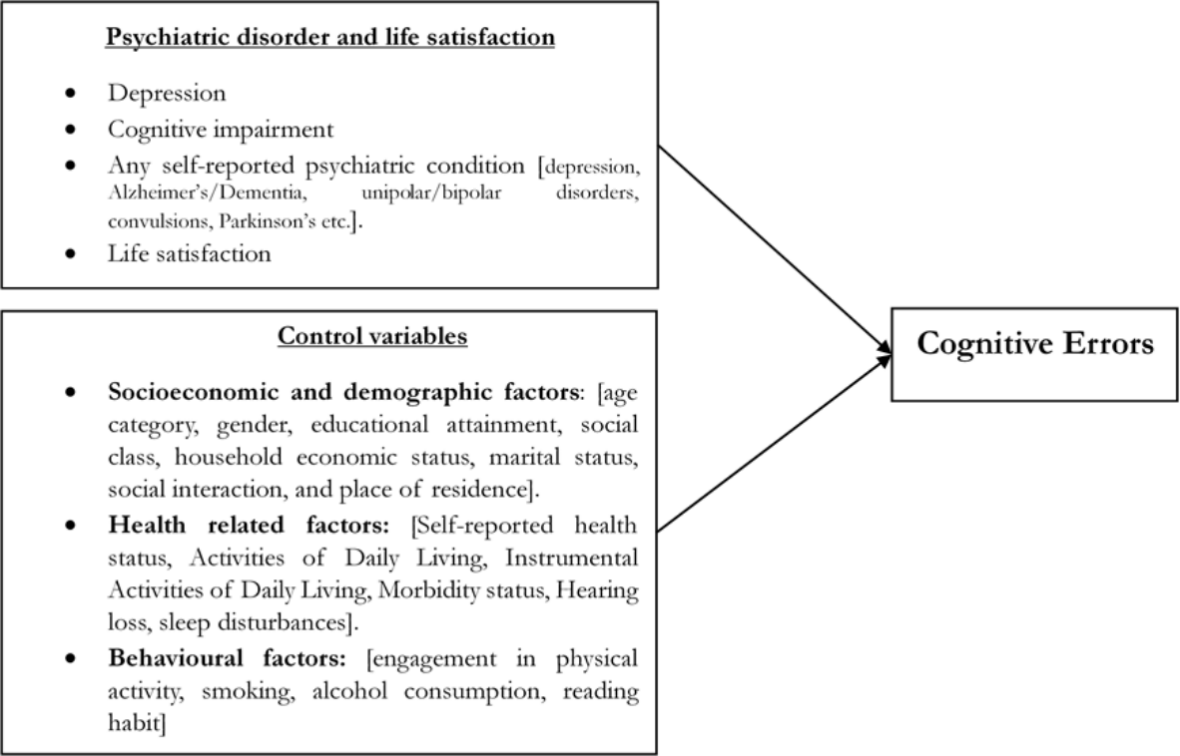
Summary of the conceptualization of cognitive errors and their predictors.

**Fig. 2:**
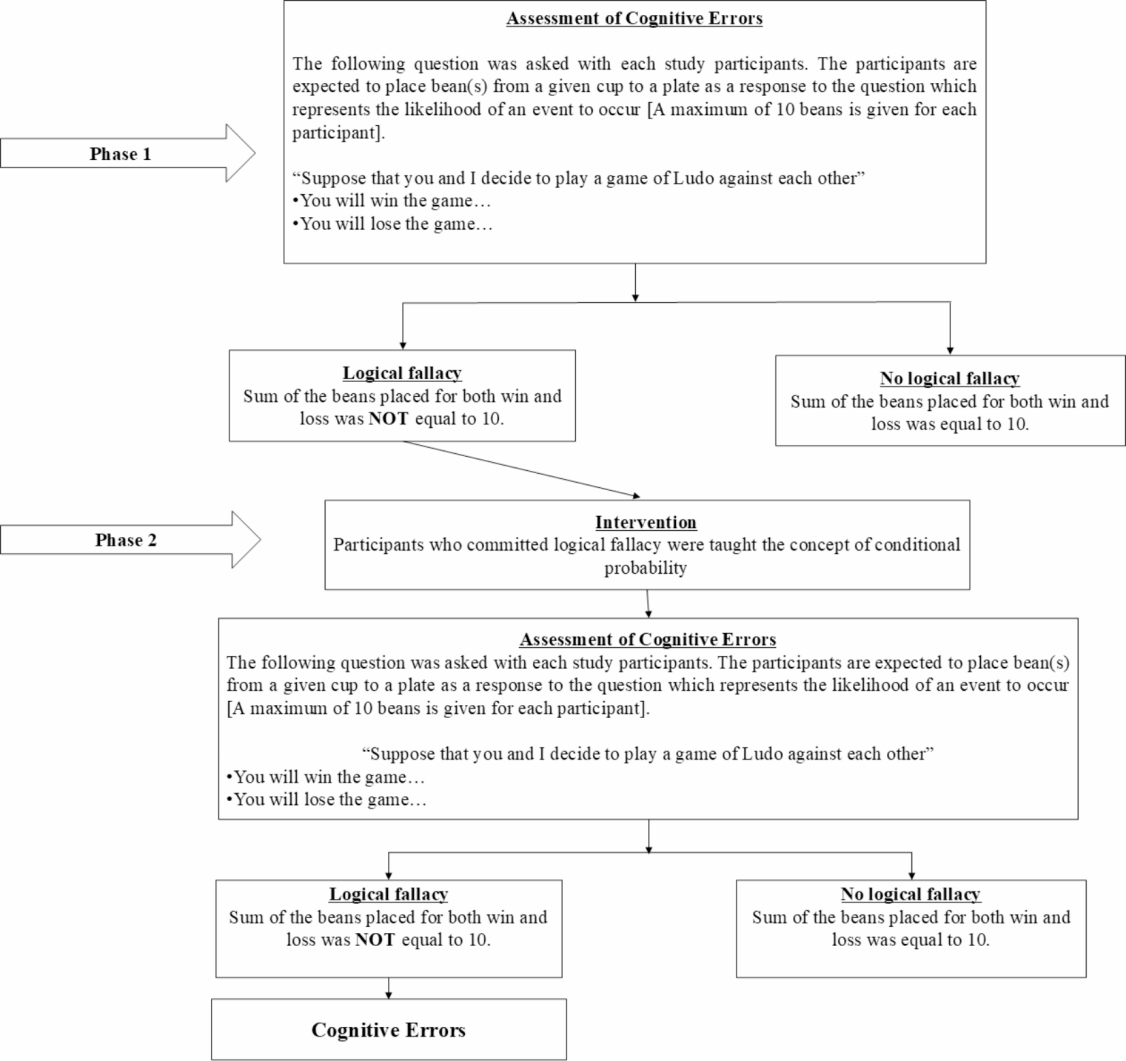
Conceptualization of cognitive errors.

This brings in the significant need to understand the determining factors of cognitive errors among middle-aged and older adults in India. The current study attempted to understand the role of depression, life satisfaction, cognitive impairment, and self-reported psychiatric/neurological conditions on cognitive errors along with covariates such as socioeconomic, demographic, health-related, behavioural risk, and other factors (the study’s conceptual framework is presented in Fig. 1). We have considered the above mentioned factors as determinants, as life satisfaction and mental health factors are generally associated with cognitive functioning and errors^44,45^. In the present study, we aimed to understand cognitive errors through an experimental approach (Refer to Fig. 2 for conceptualization of cognitive errors).

## Methods

### Data and sample

The study used unit-level data from the first wave of the Longitudinal Ageing Study in India (LASI) conducted between 2017 and 2018. The data investigate the socioeconomic, health, psychosocial components, and other aspects of the ageing population in India. The LASI was funded by the Ministry of Health and Family Welfare, the Government of India, the National Institute of Aging, and the United Nations Population Fund India. The data collection was designed and executed by the International Institute for Population Sciences (IIPS) along with technical support from various institutions, including Harvard T H Chan School of Public Health (HSPH), and the University of Southern California (USC)^46^. LASI adopted a three-stage and four-stage sampling design for rural and urban areas.

**Fig. 3:**
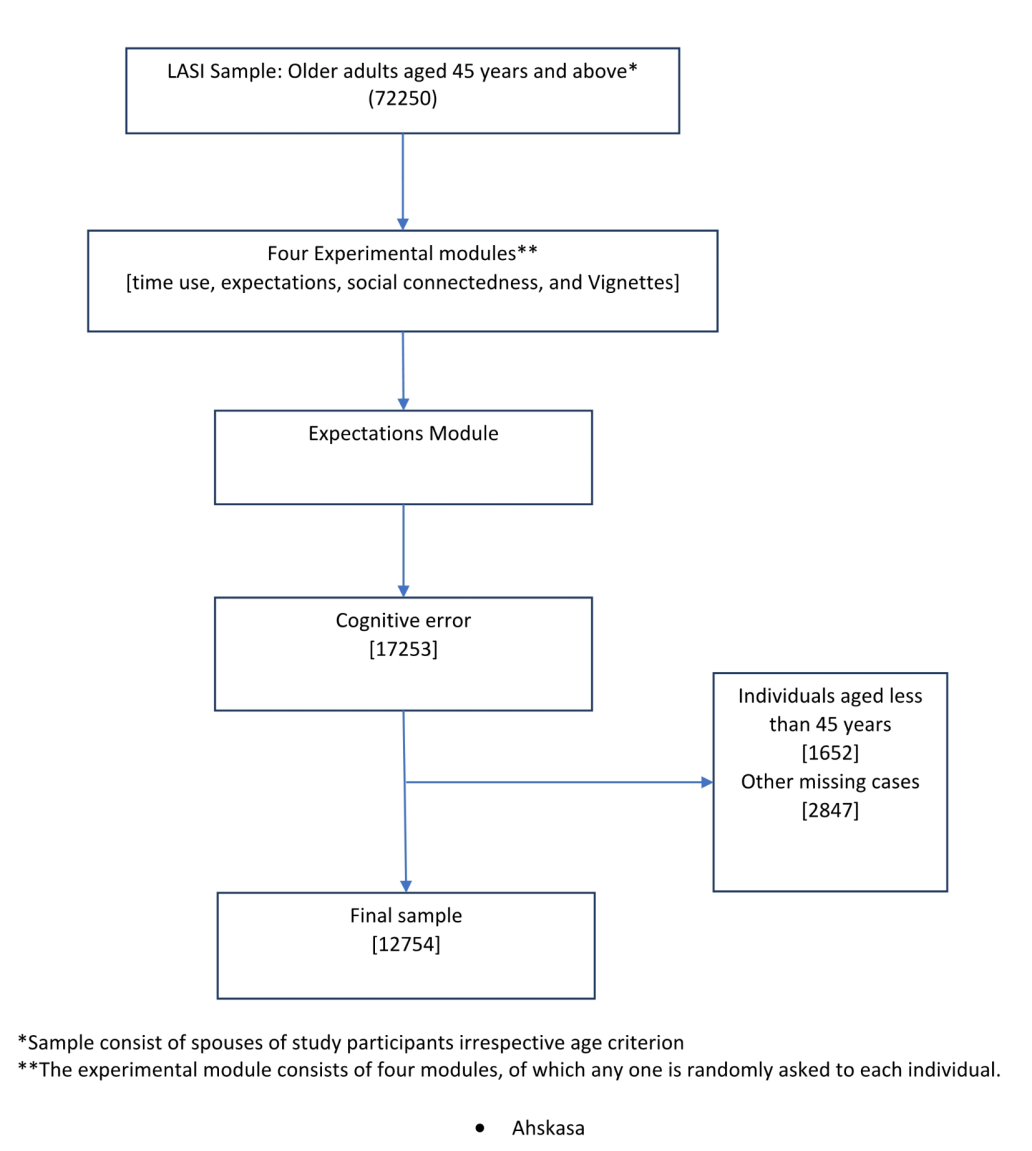
Sample selection procedure.

LASI included 72250 adults representing all Indian states and union territories (excluding Sikkim) aged 45 years and above. The current study considered data from the experimental module, consisting of four separate sections: time use, expectations, social connectedness, and vignettes. One of these sections was chosen randomly to collect data from each individual. This study initially considered 17253 individuals from the ‘expectation’ section. Further, after removing individuals below 45 years and other missing cases, the final sample included for analysis was 12,754 individuals. The summary of the sample selection process is presented in Fig. 3.

## Measures

### Conceptualization of cognitive errors

The “*Homo Economicus*” theory, widely considered the base for developing socioeconomic theories, emphasizes that humans are consistently rational, close-to-optimal agents and can solve simple and complex cognitive decision-making problems^47^. Social scientists in the late 20th century weighted the importance of “*bounded rationality*” with the support of empirical evidence asserting that humans often fail in their decision-making processes as they are not perfectly rational^48–50^. Studies indicated that the human tendency to depend on heuristic or mental shortcuts causes cognitive errors that may result in irrational decisions^51^. In social and behavioural sciences, people are considered incomplete Bayesians as they sometimes designate erroneous probability values about outcomes, as found in several recent studies^52–55^. These possibilities lead to cognitive errors.

In the present study, cognitive errors were estimated using an activity based experimental design. In the expectations module of the LASI survey, an experimental set-up was created for the study participants. Different questions were asked about the chance or the likelihood of certain events that are going to happen. For the experiment, each participant was given 10 beans in a cup and asked to choose out of them and put them onto a plate representing the likelihood of an event. The participants were further informed that adding no beans onto the plate meant the event would not happen. Adding beans increases the likelihood of the event happening. Adding ten beans onto the plate means the participant is sure that the event will happen. Additionally, the interviewer demonstrated the experiment by creating a hypothetical question about the likelihood of a cold in the coming year. In this hypothetical example, participants were informed that if the participant think about the chance that there will have a cold in the next year; if the participant put 4 beans onto the plate, it means that out of 10 people who are exactly like the participant, 4 would have a cold in the next year. It also means that 6 out of those 10 people would not have a cold in the next year. Along with this hypothetical example, the interviewer has demonstrated the case by putting 4 beans onto the plate. Once the participant understood the method of participation in the experiment, the following question was asked in the actual experiment.

“Suppose that you and I decide to play a game of Ludo against each other.”

The responses by the participants regarding the likelihood of winning and losing were collected in two phases. In the first phase, the participants were asked to pick the number of beans that reflects how likely they think it is that:

- They will win the game….
- They will lose the game….

In the second phase, the participants were taught the concept of conditional probability, and responses were collected as in the first phase. In the second phase, the participants were informed that since only two outcomes are possible at the end of the Ludo game, i.e., either win or lose, it means that they may win the game with a given probability [equivalent to the number of beans added to the plate] and the remaining is the probability of loss. Mathematically, it can be expressed as follows.

1 $$\:P\left(Win\right)=X$$

2 $$\:P\left(Loss\right)=\left(1-X\right)$$

In the above expressions (1) and (2), X represents the number of beans added to the plate. Technically, the events, win and loss, are mutually exclusive and dependent. It can be expressed as follows.

3 $$\:P\left(Win\:\cap\:Loss\right)=0$$

The cognitive errors were estimated when the participants committed “logical fallacy”. In other words, cognitive errors occurred when the participants deviated from the basic understanding of probability rules. Such deviations from the basic logic can be classified as (exclusive) “disjunction fallacy”^56^. Study participants who responded in line with the probability logic (i.e., the sum of beans placed in the plate for both win and loss was equal to ten) were considered not committed cognitive errors in the first phase. The remaining participants were taught the basic concept of conditional probability and then considered for the second phase of the experiment. For this, participants were informed that the sum of beans placed onto the place for both win and loss should be 10. We also identified participants with cognitive errors when they repeated the “logical fallacy” in the second phase. Those who did not commit such “logical fallacy” in the second phase were considered cases of non-cognitive errors. A summary of the conceptual framework of the experimental design is presented in Fig. 2.

### Predictor variables

**Depression**: Previous studies established that depression is a decisive factor that may negatively affect cognitive ability^57–59^. The present study considered depression symptoms as a primary predictor variable of cognitive errors. In this study, depression was measured using the Center for Epidemiological Studies Depression (CESD) score. A short version of the depression measurement tool (CESD-10) consisting of 7 negative and 3 positive feelings questions was employed in the survey. The total score from these 10 questions was calculated after reversing the score of the positive questions (on a 0–10 scale). We classified individuals with “depression symptoms” if the CESD score was 4 and above, and less than 4 was classified as “no depression symptoms”^60^.

**Life satisfaction**: Life satisfaction level is an established associated factor of cognitive ability. Studies indicated that life satisfaction prevents cognitive impairment risk among the ageing population^61^. Studies also indicated that life dissatisfaction is associated with the risk of dementia^62^. In the present study, the participants’ life satisfaction was assessed based on five questions in the survey. The five questions include: (i) In most ways, my life is close to ideal, (ii) The conditions of my life are excellent, (iii) I am satisfied with my life, (iv) So far, I have got the important things I want in life, and (v) If I could live my life again, I would change almost nothing. Response to each of these questions was collected on a seven scale with a value of 1 representing “strongly disagree”, 2 representing “somewhat disagree”, 3 representing “slightly disagree”, 4 representing “neither agree nor disagree”, 5 representing “slightly agree”, 6 representing “somewhat agree”, and 7 representing “strongly agree”. The scores from all five questions were combined, and a life satisfaction score ranging between 5 and 35 was created, with a higher score representing a higher level of life satisfaction. Additionally, the internal consistency and reliability was assessed and the result indicated that the scale is a good measurement tool of life satisfaction with a Cronbach Alpha value of 0.90.

**Cognitive impairment**: Studies indicated the importance of cognitive impairment and showed that individuals with cognitive impairment are at multiple risks, including the decline of functional ability, memory loss, and other psychiatric disorders^63,64^. In the present study, cognitive impairment was measured as recommended by LASI using a set of questions on memory (word recall test), orientation (time and place), arithmetic function (number counting and computation), executive function (paper folding and drawing test) and object naming^65^. The composite cognitive score ranged between 0 and 43, with a higher score for improved cognitive functions. We classified the lowest 10% as having low cognitive status^66^.

**Self-reported psychiatric/neurological conditions**: We further considered the study participants’ self-reported neurological or psychiatric problems diagnosis. We classified individuals as having neurological or psychiatric problems if they have reported a physician diagnosis of any neurological or psychiatric problems among depression, Alzheimer’s/Dementia, unipolar/bipolar disorders, convulsions, Parkinson’s, etc.

**Socioeconomic and demographic factors**: The study considered a range of covariates that could potentially affect cognitive errors. The socioeconomic and demographic factors consist of age categories (45–59 years, 60–69 years and 70 years and above), gender (male and female), education in years (no schooling, up to 5 years, 6–10 years and above 10 years), and social class, which includes Scheduled Tribes (ST), Scheduled Caste (SC), Other Backward Class (OBC) and Others. We have also considered the household economic status that was categorized as poorest, poor, middle, richer and richest; living arrangement (living alone, with spouse and others and with others); marital status (in a union and not in a union) and residency type (rural and urban). We have also included study participants’ social interaction, which could determine their cognitive ability. Social interaction was categorized as ‘yes’ if the participants visited relatives/friends and as ‘no’ if otherwise. The household economic status was measured based on the expenditure data of each household on 11 food and 29 non-food items after standardizing the expenditure to a 30-day reference period^65^.

**Health–related factors**: It was well established that health factors significantly contribute to cognitive errors^67,68^. The health-related factors include self-rated health (SRH), Activities of Daily Living (ADL), Instrumental Activities of Daily Living (IADL), and morbidity status. The response to the SRH question was categorized into “good” by combining very good, good, and fair and “poor” by combining poor and very poor. The ADL and IADL consist of six and seven types of difficulties in various aspects of everyday functioning, respectively. ADL was measured using questions on six types of difficulties in dressing, walking, bathing, eating, using the toilet, and getting out of bed. IADL was measured using seven questions on difficulties in performing cooking, shopping, making telephone calls, taking medications, doing work around the house/gardening, managing money, and movement. ADL and IADL were classified as “low” if the difficulty was reported in performing at least one of the ADL and IADL items, respectively^69^. The morbidity status was measured based on the presence of some diseases as reported by the participants among the nine diseases listed; the responses were classified as “no disease”, “one disease”, and “multimorbidity”. The major diseases considered include hypertension, diabetes, cancer, chronic lung diseases, chronic heart diseases, stroke, arthritis, any neurological or psychiatric issues, and high cholesterol. In addition, we have considered hearing loss and sleep disturbances among the study participants. Hearing was assessed based on any hearing problem reported among the study participants. Sleep disturbances of the study participants were measured based on the Jenkin’s Sleep Scale (JSS) and considered a continuous variable. The measure indicated a good reliability (Cronbach’s Alpha = 0.82).

**Behavioural-risk factors**: The literature suggests that involvement in behavioural risk factors such as smoking and drinking alcohol, in the long run, impairs cognitive abilities later in life^70,71^. The current study considers alcohol consumption (yes and no), smoking (yes and no), and involvement in physical activity (yes and no) as behavioural risk factors.

**Other factors: **Studies indicated that reading habits reduce cognitive impairment^72^ and prevent cognitive decline among older adults^73^. This study includes a component about reading habits from the social activities section of the LASI data. The reading habit was assessed using the survey question, ‘How often, if at all, do you read books/newspapers/magazines?’ and the responses were classified into ‘daily readers, ‘read sometimes’, and ‘never readers’.

### Statistical analysis

In the present study, we conducted descriptive statistics and bivariate analysis to report the characteristics of the study sample. Further, multivariable logistic regression was employed to estimate the odds of committing cognitive errors on respondents’ background characteristics. First, we examined the association between depression, life satisfaction, cognitive impairment, and self-reported psychiatric/neurological conditions with cognitive errors independently and estimated the crude odds ratio. We have considered other confounding variables in the full model (and estimated the adjusted odds ratio), including socioeconomic and demographic factors, health-related factors, behavioural risk factors, and others. The results were presented as odds ratio, and 90%, 95%, and 99% confidence intervals were considered for drawing conclusions. All the statistical analysis was performed using Stata Version 16.

## Results

The study tries to uncover the factors determining cognitive errors among ageing adults in India. The main factors considered in the study are depression, life satisfaction, cognitive impairment, and neurological conditions, along with covariates such as socioeconomic and demographic factors, health-related factors, behavioural risk factors, and other factors like reading.

The descriptive characteristics of the study variables are presented in Table 1 (*N* = 12754). The results indicated that 27.49% of adults had depression, 11.44% had cognitive impairment, and about 2% had neurological conditions. The mean score on life satisfaction was 23.93. More than 50% of the study participants were aged 45–59 years old, and nearly 52% of the total sample were females. It was found that 45.37% of participants had no formal schooling. The major proportion of the sample belonged to OBC social background (44.76%), and an approximately equal proportion of older adults belonged to different categories of household economic status: poorest (20.79%), poorer (21.26%), middle (20.65%), richer (18.58) and richest (18.73%). In addition, about 76.18% of the study participants were in a marital union. A majority of the study participants were rural residents (67.98%). About 16.33% reported poor self-reported health status. About 15% and 35% of the sample reported low ADL and IADL statuses, respectively. More than half of the participants had no morbidity conditions (53.95%). About 57% of the participants did not engage in physical activity (57.05%). It was found that 38.09% and 15.13% were ever smokers and consumers of alcohol. Just above 15% of the study participants reported being daily readers.

**Table 1:** Descriptive characteristics of the study variables.

Variable	Number of cases	W%
Depression symptom		
No	9595	72.51
Yes	3159	27.49
Life satisfaction score (mean)	12,754	23.93
Cognitive impairment		
No	11,477	88.56
Yes	1277	11.44
Neurological condition		
No	12,505	97.99
Yes	249	2.01
Age category		
45–59 years	6943	52.53
60–69 years	3707	29.11
70 years and above	2104	18.36
Gender		
Male	6183	47.85
Female	6571	52.15
Educational attainment		
No schooling	5388	45.37
Up to 5 years	2427	18.43
6–10 years	3384	23.25
Above 10 years	1555	12.95
Social class		
Scheduled Tribe	2048	7.83
Scheduled Caste	2159	19.65
Other Backward Class	4796	44.76
Others	3751	27.76
Household economic status		
Poorest	2381	20.79
Poorer	2565	21.26
Middle	2542	20.65
Richer	2594	18.58
Richest	2672	18.73
Marital status		
In a union	9773	76.18
Not in a union	2981	23.82
Social interaction		
Yes	10,641	82.12
No	2113	17.88
Place of residence		
Rural	8026	67.98
Urban	4728	32.02
Self-reported health status		
Good	10,786	83.67
Poor	1968	16.33
Activities of Daily Living (ADL)		
High	11,173	85.61
Low	1581	14.39
Instrumental Activities of Daily Living (IADL)		
High	8872	65.28
Low	3882	34.72
Morbidity status		
None	6807	53.95
One disease	3580	27.92
Multimorbidity	2367	18.13
Hearing loss		
No	11,899	93.53
Yes	855	6.47
Engagement in vigorous physical activity		
No	7485	57.05
Yes	5269	42.95
Ever smoker		
No	7977	61.91
Yes	4777	38.09
Ever consumed alcohol		
No	10,426	84.87
Yes	2328	15.13
Reading habit		
Daily	2187	15.67
Some times	2228	15.68
Never	8339	68.65
Total	12,754	100

W%: weighted percentage.

Table 2 indicates the bivariate analysis of cognitive errors with depression, cognitive impairment, neurological condition, and other baseline characteristics. The results indicated that ageing adults with depression symptoms had committed cognitive errors than those with no depression symptoms (26.75% Vs 21.12, *p* < 0.01). It was also revealed that participants who reported cognitive impairment had a higher prevalence of cognitive errors than participants with no cognitive impairment (30.00% Vs 21.72%, *p* < 0.01). Similarly, it was found that cognitive errors were high among participants who reported any neurological condition in comparison with those who did not report any neurological condition (36.41% Vs 22.39%, *p* < 0.01).

**Table 2:** Bivariate analysis of cognitive errors with psychiatric factors, life satisfaction and other background characteristics (Chi-squared test).

Independent variable	Percentage (w%)	*P* value
Depression symptom		
No	21.12	< 0.01
Yes	26.75	
Cognitive impairment		
No	21.72	< 0.01
Yes	30.00	
Neurological condition		
No	22.39	< 0.01
Yes	36.41	
Age category		
45–59 years	21.70	0.04
60–69 years	22.87	
70 years	25.11	
Gender		
Male	21.70	0.02
Female	23.56	
Educational attainment		
No schooling	25.17	< 0.01
Up to 5 years	22.11	
6–10 years	19.56	
Above 10 years	20.30	
Social class		
Scheduled Tribe	18.37	< 0.01
Scheduled Caste	26.72	
Other Backward Class	23.54	
Others	19.62	
Household economic status		
Poorest	21.15	0.91
Poorer	22.71	
Middle	23.33	
Richer	23.25	
Richest	23.02	
Marital status		
In a union	21.99	< 0.01
Not in a union	24.85	
Social interaction		
Yes	22.00	< 0.01
No	25.74	
Place of residence		
Rural	23.28	0.04
Urban	21.38	
Self-reported health status		
Good	21.97	0.24
Poor	26.25	
Activities of Daily Living (ADL)		
High	22.42	0.88
Low	24.13	
Instrumental Activities of Daily Living (IADL)		
High	20.41	< 0.01
Low	26.92	
Morbidity status		
None	21.82	0.25
One disease	21.23	
Multimorbidity	27.40	
Hearing loss		
No	22.54	0.30
Yes	24.51	
Engagement in vigorous physical activity		
No	23.72	< 0.01
Yes	21.27	
Ever smoker		
No	23.82	< 0.01
Yes	20.79	
Ever consumed alcohol		
No	22.54	0.17
Yes	23.38	
Reading habit		
Daily	18.36	< 0.01
Some times	22.14	
Never	23.78	
Total	22.67	

W%: weighted percentage, *P* value is based Chi-squared test.

**Fig. 4:**
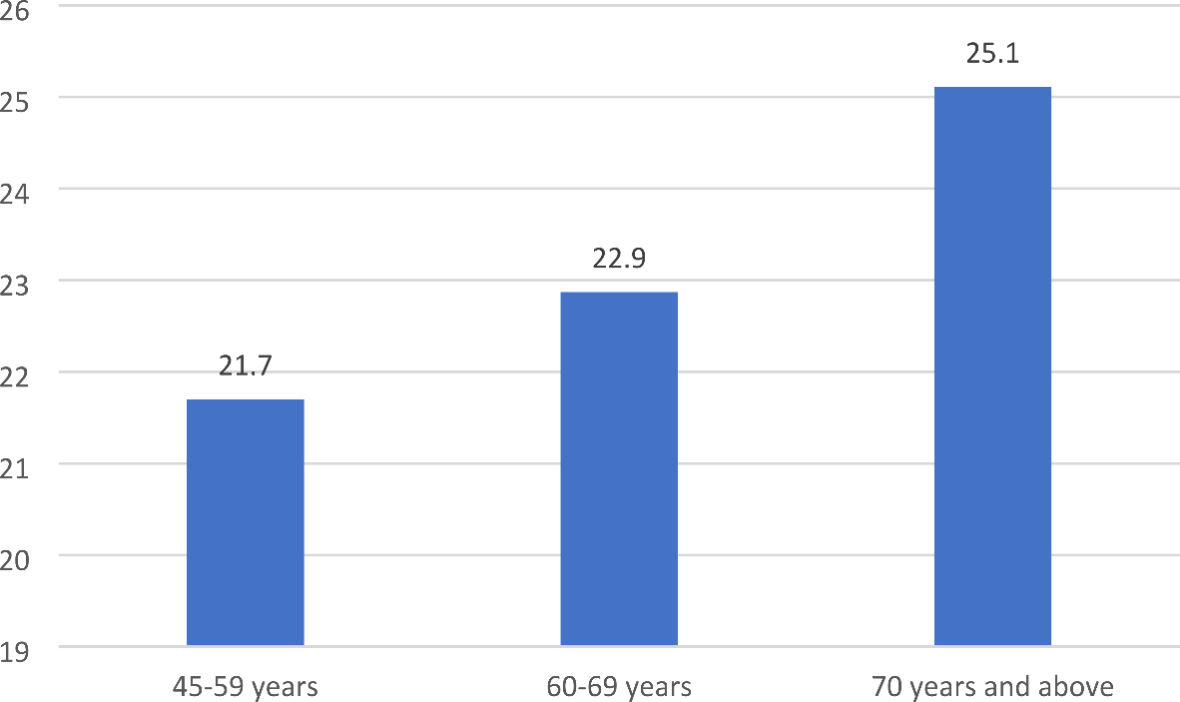
Prevalence of cognitive errors (%) by age groups.

The prevalence of cognitive errors increased with age and was significantly higher among older adults aged 70 years and above compared to those aged 45–59 years (25.1% Vs 21.70%, *p* < 0.05) (Fig. 4). In addition, the bivariate analysis in Table 2 indicated that female participants committed higher cognitive errors than their counterparts (23.56% Vs 21.70%, *p* < 0.05). The cognitive errors significantly differed across educational attainment categories, with higher prevalence among individuals with no schooling and lower prevalence among participants with more years of schooling. Cognitive errors were higher amongageing from SC backgrounds. Based on marital status, it was found that participants who were in a marital union had a lower prevalence of cognitive errors compared to their counterparts (21.99% Vs 24.85%, *p* < 0.01). Similarly, cognitive errors were lower among participants who maintained social interaction than their counterparts (22.00% Vs 25.74%, *p* < 0.01). Also, individuals in urban areas had a lower prevalence of cognitive errors than those in rural areas (21.38% Vs 23.28%, *p* < 0.05). It was further indicated that cognitive errors were significantly lower among participants with high IADL status and those who engaged in regular physical activities and reading.

The results of multivariate logistic regression of cognitive errors with depression, life satisfaction, cognitive impairment, neurological condition, and other background characteristics are presented in Table 3. In the unadjusted model, the results indicated that adults with depression symptoms (cOR = 1.45, 99% CI: 1.32–1.58), cognitive impairment (cOR = 1.31, 99% CI: 1.15–1.49), and neurological condition (cOR = 1.44, 99% CI: 1.09–1.88) had higher odds of committing cognitive errors compared to their respective counterparts. Similarly, in the unadjusted model, individuals with higher life satisfaction had a significantly lower likelihood of committing cognitive errors (cOR = 0.99, 99% CI: 0.98–0.99). In the fully adjusted model, the results for all these variables showed a consistent result, with higher odds of depression symptoms (aOR = 1.28, 99% CI: 1.16–1.41), cognitive impairment (aOR = 1.13, 90% CI: 1.00–1.30) and higher likelihood with life satisfaction (aOR = 0.99, 99%, CI: 0.98–1.00). Participants with neurological conditions had higher odds of committing cognitive errors, but this was not statistically significant.

**Table 3:** Results of multivariable logistic regression of cognitive errors with psychiatric factors, life satisfaction and other background characteristics.

	Cognitive error		Cognitive error		Cognitive error		Cognitive error		Cognitive error	
Variables	Crude odds ratio (cOR) (CI)	*P* value	Crude odds ratio (cOR) (CI)	*P* value	Crude odds ratio (cOR) (CI)	*P* value	Crude odds ratio (cOR) (CI)	*P* value	Adjusted odds ratio (aOR) (CI)	*P* value
Depression symptom (Ref: No)										
Yes	1.45	< 0.001							1.28	< 0.001
	(1.32–1.58)								(1.16–1.41)	
Life satisfaction score			0.99	< 0.001					0.99	0.002
			(0.98–0.99)						(0.98–1.00)	
Cognitive impairment (Ref: No)					1.31	< 0.001			1.13	0.090
Yes					(1.15–1.49)				(1.00–1.30)	
Self-reported psychiatric/neurological conditions (Ref: No)										
Yes							1.44	0.009	1.22	0.179
							(1.09–1.88)		(0.91–1.61)	
Age in years									1.00	0.596
									(0.99–1.00)	
Gender (Ref: Female)										
Male									1.16	0.006
									(1.04–1.28)	
Educational attainment (Ref: No schooling)										
Up to 5 years									0.94	0.292
									(0.83–1.06)	
6–10 years									0.89	0.078
									(0.78–1.00)	
More than 10 years									0.87	0.117
									(0.72–1.04)	
Social class (Ref: Scheduled Tribe)										
Scheduled Caste									0.77	< 0.001
									(0.68–0.89)	
Other backward class									0.73	< 0.001
									(0.63–0.82)	
Others									0.57	< 0.001
									(0.50–0.65)	
Household economic status (Ref: Poorest)										
Poorer									1.13	0.066
									(1.00–1.29)	
Middle									1.16	0.031
									(1.01–1.32)	
Richer									1.21	0.006
									(1.06–1.38)	
Richest									1.24	0.002
									(1.08–1.43)	
Marital status (Ref: In a union)										
Not in a union									1.09	0.011
									(1.00–1.21)	< 0.001
Social interaction (Ref: Yes)										
No									1.28	< 0.001
									(1.15–1.43)	
Place of residence (Ref: Rural)										
Urban									0.81	< 0.001
									(0.74–0.89)	
Self-reported health status (Ref: Good)										
Poor									0.89	0.063
									(0.79–1.00)	
Activities of Daily Living (Ref: High)										
Low									0.81	0.003
									(0.70–0.93)	
Instrumental Activities of Daily Living (Ref: High)										
Low									1.24	< 0.001
									(1.12–1.37)	
Morbidity Status (Ref: None)										
One disease									0.98	0.781
									(0.89–1.09)	
Multi morbidity									1.00	0.931
									(0.89–1.13)	
Hearing loss (Ref: No)										
Yes									1.02	0.799
									(0.87–1.20)	
Sleep disturbances									1.03	< 0.001
									(1.02–1.04)	
Engagement in vigorous physical activity (Ref: No)										
Yes									0.85	0.001
									(0.78–0.93)	
Ever smoker (Ref: No)										
Yes									0.78	< 0.001
									(0.71–0.86)	
Ever consume alcohol (Ref: No)										
Yes									0.98	0.814
									(0.87–1.11)	
Reading habit (Ref: Daily)										
Some times									1.36	< 0.001
									(1.17–1.58)	
Never									1.12	
									(0.97–1.30)	0.129
Constant	0.29	< 0.001	0.46	< 0.001	0.31	< 0.001	0.32	< 0.001	0.39	< 0.001
	(0.28–0.30)		(0.40–0.53)		(0.30–0.32)		(0.31–0.33)		(0.27–0.56)	
Sample	12,754		12,754		12,754		12,754		12,754	

Confidence Intervals in parentheses.

**Fig. 5:**
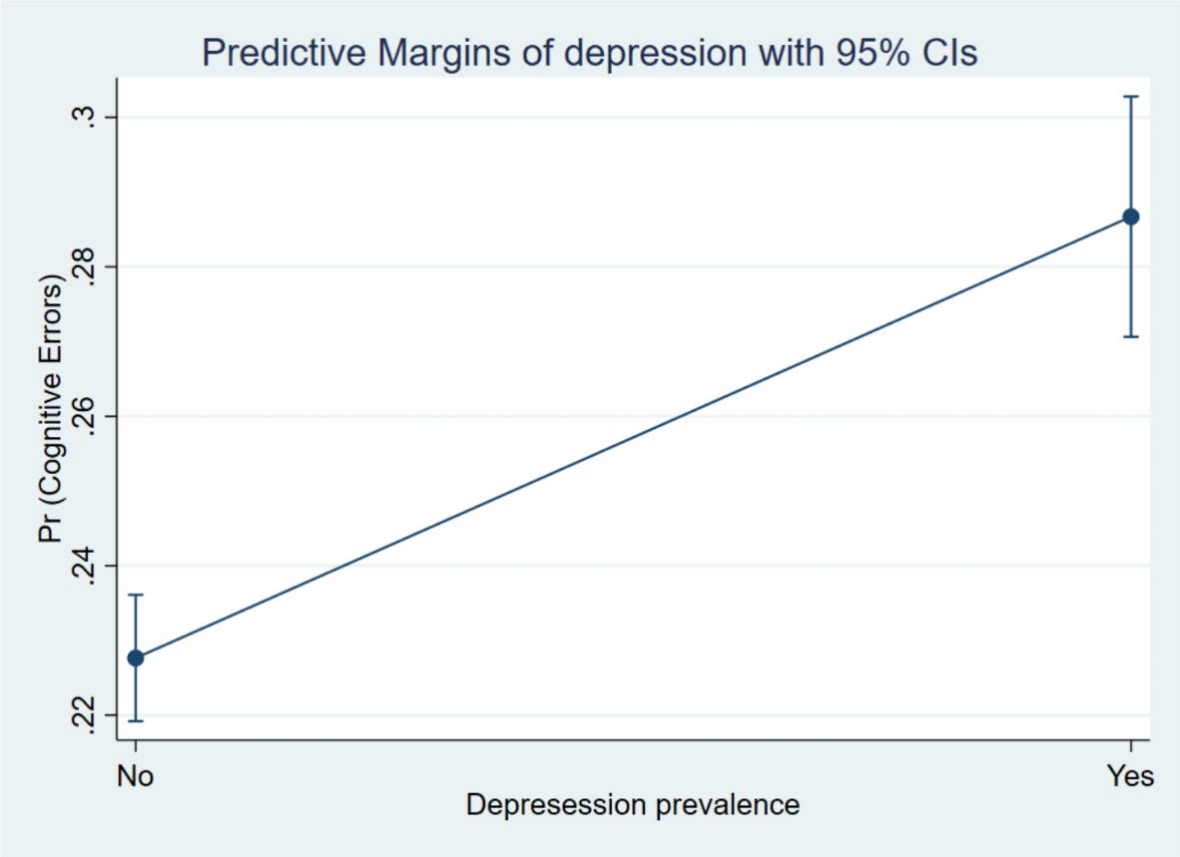
Margin plot representing the probabilities of cognitive errors with depression prevalence status.

**Fig. 6:**
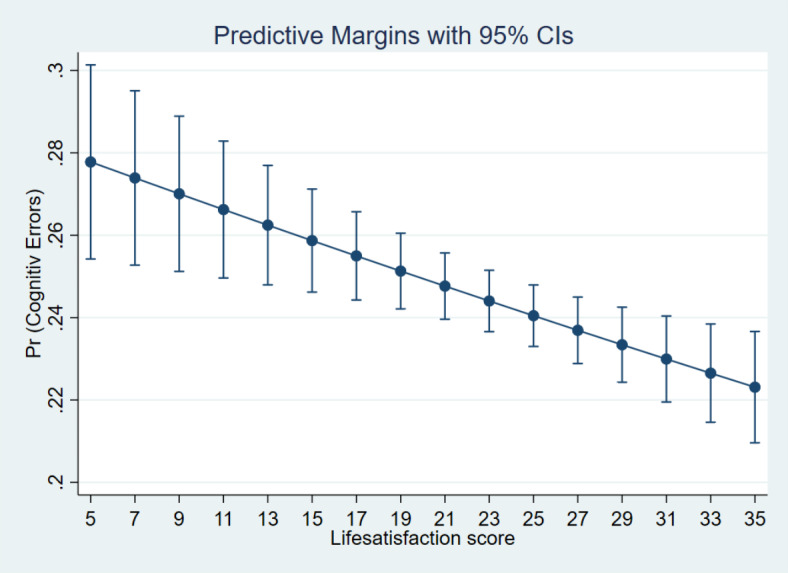
Margin plot representing the probabilities of cognitive errors with life satisfaction score.

**Fig. 7:**
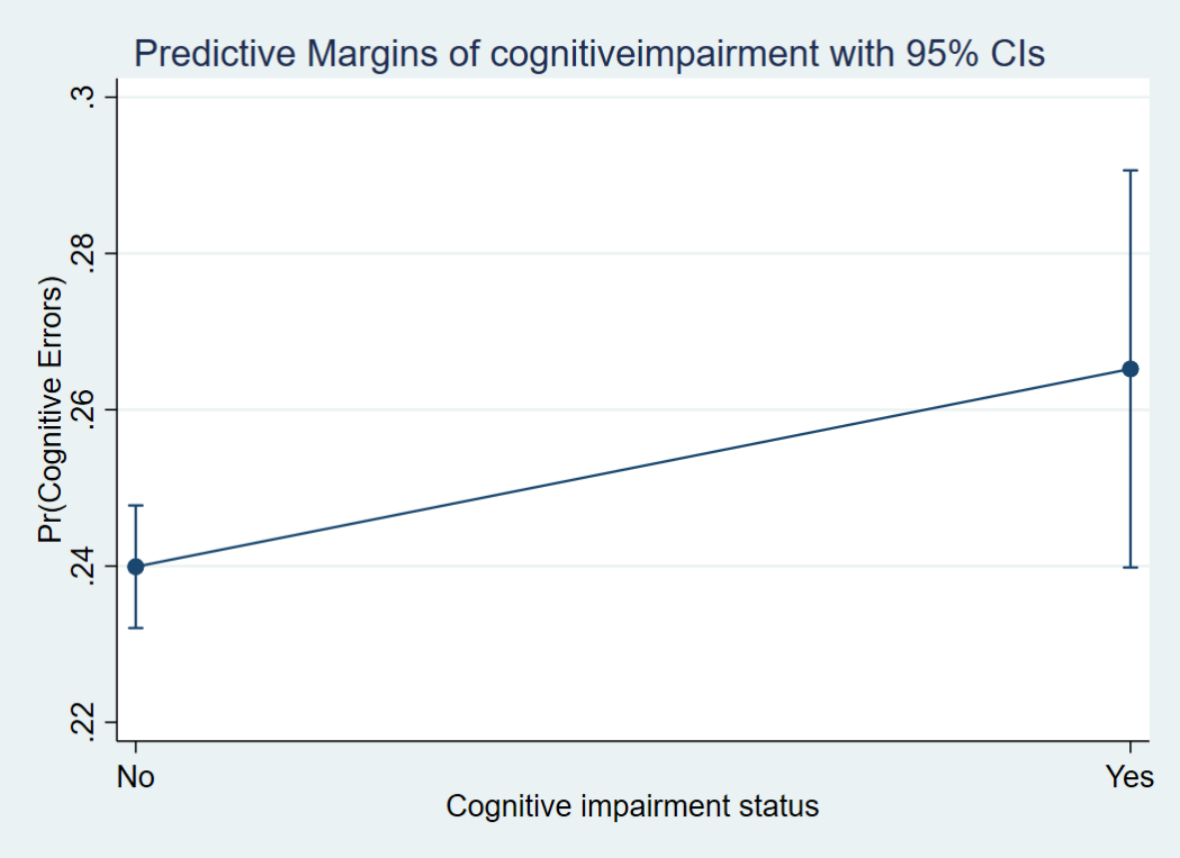
Margin plot representing the probabilities of cognitive errors with cognitive impairment status.

**Fig. 8:**
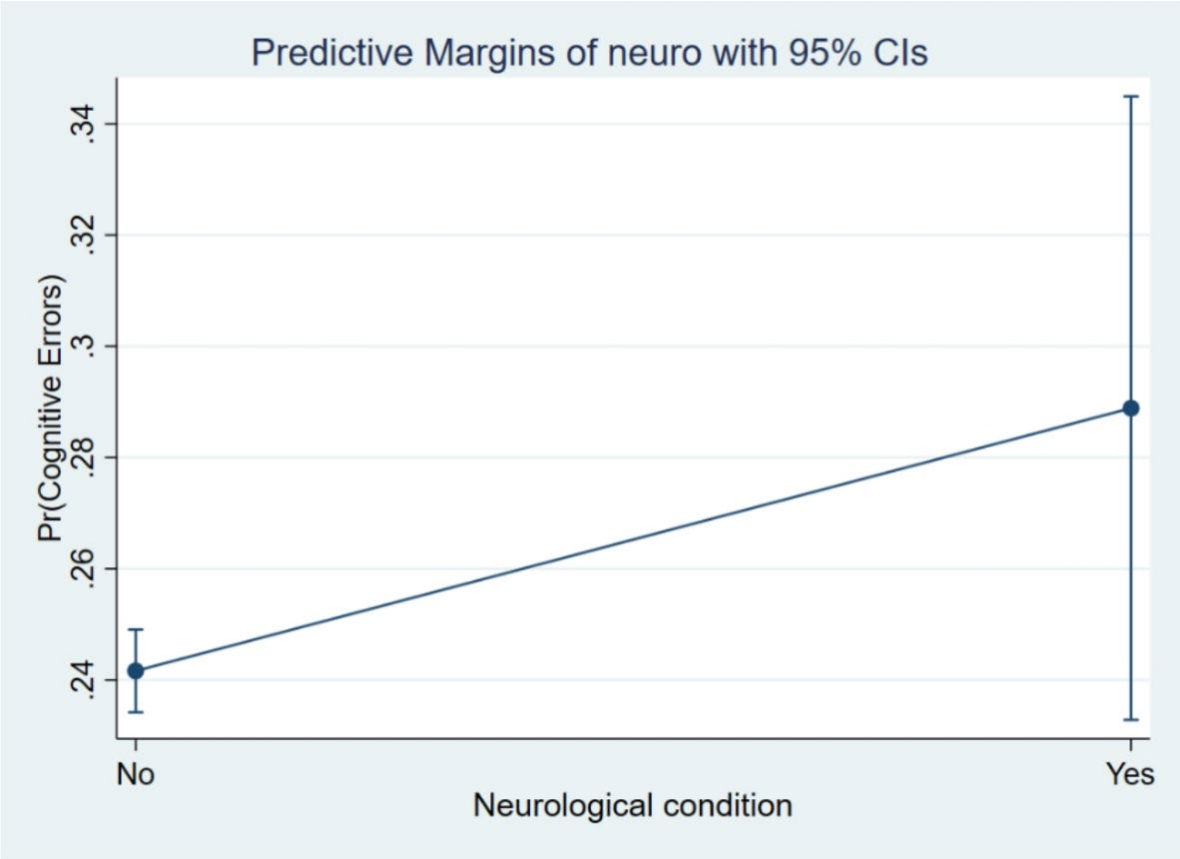
Margin plot representing the probabilities of cognitive errors with self-reported psychiatric/neurological conditions.

Additionally, the margin plots representing the probabilities of cognitive errors by these main variables of interest are presented in Figs. 5, 6, 7 and 8. It indicated a higher probability of cognitive errors among participants with depression symptoms, cognitive impairment, and neurological conditions and lower life satisfaction scores.

In terms of gender, males (aOR = 1.16, 99% CI: 1.04–1.28) had higher odds of committing cognitive errors compared to their female counterparts. Compared to participants with no formal schooling, those with more than 6–10 years of education had lower cognitive errors (aOR = 0.89, 90% CI: 0.78–1.00). Individuals from Schedules Caste, Other Backward Classes, and other social classes had lower odds of committing cognitive errors than those from the ST social class (aOR = 0.77, 99% CI: 0.68–0.89, aOR = 0.73, 99% CI: 0.63–0.82, and aOR = 0.57, 99% CI: 0.50–0.65, respectively). It was found that ageing adults from higher household economic status had higher odds of cognitive errors. In addition, individuals who were not in a marital union had a higher odd of cognitive errors than those who were in a marital union (aOR = 1.09, 95% CI: 1.00–1.21). Similarly, it was found that individuals with no social interaction had a higher odds of cognitive errors than individuals who maintained social interaction (aOR = 1.28, 99% CI: 1.15–1.43). It was also found that participants from urban areas had lower odds of cognitive errors than those from rural areas (aOR = 0.81, 99% CI: 0.74–0.89).

Based on health status, participants with low ADL status had a lower odds of cognitive errors in comparison with participants with high ADL status (aOR = 0.81, 99% CI: 0.70–0.93), whereas older adults with low IADL status had a higher odds of committing cognitive errors (aOR = 1.24, 99% CI: 1.12–1.37) in comparison with those with a high IADL status. Further, results showed that participants who were involved in regular physical activities (aOR = 0.85, 99% CI: 0.78–0.93) had lower odds of committing cognitive errors than those who did not engage in regular physical activities. It was found that participants who reported hearing loss had a higher risk of cognitive errors, but this was not statistically significant. Sleep disturbances were a significant predictor of cognitive errors, and an increase in sleep disturbances was associated with a higher likelihood of cognitive errors. The results showed that smokers had lower odds of committing cognitive errors than non-smokers. The results indicated that compared to daily readers, those engaged in reading sometimes had higher odds of cognitive errors (aOR = 1.36, 99% CI: 1.17–1.58).

## Discussion

The present study, with a range of physical and psychological variables, tried to understand the predictors of cognitive errors among middle-aged and older adults in India. The findings, consistent with the literature^23^, suggest that depression, reduced life satisfaction, cognitive impairment, and neurological conditions lead to a higher risk of committing cognitive errors. A study suggested that the effectiveness of decision-making was reduced due to the failure to use effective strategies to decide among depressed individuals^22^, leading to higher cognitive errors^74^. Limited studies examine cognitive errors as an outcome of poor life satisfaction. Earlier studies have explored the other way by exploring the role of cognitive failures and emotional distress or loneliness on life satisfaction^21,75^.

In addition, a study among older adults with bipolar disorder suggested that cognitive failures, directly and indirectly, affected their quality of life^76^. Interestingly, these factors are interconnected, where depression could lead to cognitive impairment^77^ and neurological conditions impacting life satisfaction and vice-versa. Further, all these factors increase the odds of cognitive errors. In the present study, older adults who could not complete the experimental task appropriately, even after the prompt of conditional probability, may need attention in terms of neurological and psychological interventions. A study among adults with Alzheimer’s and mild cognitive impairment revealed that committing errors in a task reduced from the previous trial with correction than individuals who were not corrected, indicating adaptive control among participants^78^.

The present study indicated that females were involved in committing higher cognitive errors than their male counterparts. In support of our findings, a previous study showed significant gender differences in the decision-making process^79^, and females were more prone to commit risky decisions, especially when they are under stereotype threat^80^. Consistent with our findings, a study among older Japanese adults showed gender differences in cognitive decline and supported the cognitive reserve hypothesis through the role of education, intellectual experiences, and occupation^81^. Interestingly, educational attainment acted as a reserve and reduced the risk of committing cognitive errors in this study. In addition, a systematic review indicated the impeccable role of education on cognition across the lifespan^36^.

In addition, the risk of committing cognitive errors differed according to social class, economic status, marital status, and residence type. A previous study established that social context significantly shapes decision-making^82^. Further, a study suggested the differences in decision-making tendencies in the prestige-money games among the upper class and lower class^83^. The higher risk of cognitive errors was evident among participants from scheduled tribes might be due to various reasons, including the low level of education because, in this study, education is found to be a reserve against cognitive errors. Moreover, it is established that socioeconomic disadvantage is a risk factor for children’s cognitive development, implying its impact on life course^84^. Also, studies have documented that social disadvantages significantly impact health-related quality of life^85^, along with brain health and cognitive decline^86^. However, further investigations are required to establish the determinants of a higher risk of cognitive errors among the scheduled tribes.

In line with the present study, earlier studies suggested that being in a marital union and urban residency promoted cognitive abilities^87,88^ and reduced cognitive decline^89^ among older adults. These indirectly point to the role of decision-making and cognitive errors, as there is a relationship between cognitive abilities and decision-making^90^. In line with the study evidence, literature evidence indicates that social interaction delays cognitive decline among middle-aged and older adults^91,92^. In the present study, individuals with higher ADL and lower IADL had higher odds of committing cognitive errors. ADL assesses the basic functioning of daily living, including walking, dressing, and other activities that do not require higher cognitive abilities. However, IADL includes items such as making a telephone call and managing money, which require cognitive abilities. A study evidenced the impact of cognitive errors on disability among chronic lower back pain patients, and overgeneralization was the common cognitive error^93^. Also, functional limitations strongly predicted cognitive impairment among older adults^94^. In corroboration with the evidence from the study, a study conducted among the ageing population in developing countries indicated that perceived sleep quality was a significant contributor to cognitive performance^95^. Studies have also shown that educational attainment and involvement in physical activity can enhance cognitive performance^33,96^ and reduce cognitive decline in later life^97^. Usually, smoking is associated with poor cognitive performance^98,99^. However, this study’s results suggested that smoking reduced the risk of committing cognitive errors. Further, longitudinal studies might be needed to establish this association and its causes.

This study’s results support the cognitive bias theory developed by Tversky and Kahneman^14^, which suggests that individuals are not rational beings involved in decision-making and disobey logical rules, leading to fallacies. Further, we interpret the results to defy the concept of ‘Homo Economicus’ from the economic stand that considers human beings to be rational agents, optimizers and endure in their self-interest optimally to enhance well-being with the constraints faced^100^. Although individuals are expected to make rational choices every day, the outcome of these decisions is not always rational and logical due to the inbuilt heuristics. Further, we evidenced from the results that the psychosocial and health-related factors and sociodemographic variables contributed to these cognitive biases or errors.

The study results have important implications in the clinical context as they facilitate the identification of cognitive errors and their risk factors among ageing adults. This is especially crucial for providing holistic and integrated care for ageing adults. This implies the relevance of assessing psychiatric and behavioural health factors in primary health care provisions so early identification and interventions can be initiated. Further, considering cognitive errors among ageing adults, identification can facilitate better medical decision-making as it might interfere with their medical choices. In addition, the results promote the development of community programs and interventions to reduce cognitive errors and facilitate active ageing among adults by involving them in physical and social activities and improving their intellectual skills. The intellectual skills can be enhanced through context-specific adult literacy programs. We suggest that policymakers consider elevating intellectual skills and improving awareness of these basic prospects of human functioning. Future studies can focus on understanding these variables’ cause-effects through longitudinal designs and identify the cultural underpinning of cognitive biases and the regional differences in the global and Indian context. In addition, the intervention-based study would help develop and validate context-specific literacy and awareness programs for adults.

## Limitations

Though this study has the strength of having a large sample size and is the first to conceptualize factors associated with committing cognitive errors among Indian adults, it has certain limitations. Firstly, the study design is cross-sectional, so the cause-and-effect relationship between the variables cannot be established. The findings only indicate the association between the variables. Secondly, the study has self-reported information regarding SRH and morbidity status. Even though the self-reported variables act as a good measure, they may be prone to biases. Third, this study does not consider cultural and regional differences in terms of cognitive errors. Future studies may use intervention-based approaches and longitudinal data.

## Conclusion

The study is one of its kind and ponders the predictive factors of cognitive errors among Indian adults. Even though cognitive errors are part of everyday life, they add to the burden of ageing and are surprisingly even associated with negative psychological outcomes. This brings to the essentiality of the understanding of the determining factors of cognitive error. The study results evidenced that depression, life satisfaction, cognitive impairment, and neurological conditions, along with sociodemographic and health-related covariates, were associated with cognitive errors. It was noted that educational attainment, involvement in physical and social activities, and good quality sleep acted as reserves to inhibit and reduce cognitive errors among adults. The study results highlighted the need to enhance life satisfaction and involve adults in intellectual and physical activities, as these might delay and reduce cognitive impairment and cognitive errors. Future studies may focus on the causal relationship between these variables and developing intervention programs involving these predictive factors of cognitive errors.

## Data Availability

The data used for this study is available through the following website. https://www.iipsindia.ac.in/content/lasi-wave-i.

## References

[1] Allaire, J. C. & Gamlado, A. A. Everyday Cognition. In *Encyclopedia of Geropsychology* (ed Pachana, N. A.) 1–7 (Springer, Singapore, 2015). 10.1007/978-981-287-080-3_252-1.

[2] Ward, E. V., Dhami, M. K. & Editorial The aging decision-maker: Advances in understanding the impact of cognitive change on decision-making. *Front. Psychol.* **7**, (2016). 10.3389/fpsyg.2016.01622 PMC5073209 27818639

[3] Albarracín, D. & Wyer, R. S. The cognitive impact of past behavior: Influences on beliefs, attitudes, and future behavioral decisions. *J. Pers. Soc. Psychol.* **79**, 5–22 (2000). 10909874 10.1037//0022-3514.79.1.5 PMC4807731

[4] Spiegel, T. Influences of personal experience in decision-making. In *Exploring the Influence of Personal Values and Cultures in the Workplace* 76–97 (Business Science Reference/IGI Global, Hershey, PA, US, 2017). 10.4018/978-1-5225-2480-9.ch005.

[5] Acciarini, C., Brunetta, F. & Boccardelli, P. Cognitive biases and decision-making strategies in times of change: A systematic literature review. *Manag. Decis.* **59**, 638–652 (2020).

[6] Paulus, D., de Vries, G., Janssen, M. & Van de Walle, B. The influence of cognitive bias on crisis decision-making: Experimental evidence on the comparison of bias effects between crisis decision-maker groups. *Int. J. Disaster Risk Reduct.* **82**, 103379 (2022).

[7] Weller, J., Ceschi, A., Hirsch, L., Sartori, R. & Costantini, A. Accounting for individual differences in decision-making competence: Personality and gender differences. *Front. Psychol.* **9**, (2018). 10.3389/fpsyg.2018.02258 PMC6276324 30534098

[8] Berthet, V., Autissier, D. & de Gardelle, V. Individual differences in decision-making: A test of a one-factor model of rationality. *Personal. Individ. Differ.* **189**, 111485 (2022).

[9] Dietrich, C. Decision making: Factors that influence decision making, heuristics used, and decision outcomes. *Inq. J.* **2**, (2010).

[10] Skagerlund, K., Forsblad, M., Tinghög, G. & Västfjäll, D. Decision-making competence and cognitive abilities: Which abilities matter? *J. Behav. Decis. Mak.* **35**, e2242 (2022).

[11] Dale, S. Heuristics and biases: The science of decision-making. *Bus. Inf. Rev.* **32**, 93–99 (2015).

[12] Laguna, M. & Martí, R. Heuristics. In *Encyclopedia of Operations Research and Management Science* (eds Gass, S. I. & Fu, M. C.) 695–703 (Springer US, 2013). 10.1007/978-1-4419-1153-7_1184.

[13] Korteling, J. E., Brouwer, A. M. & Toet, A. A neural network framework for cognitive bias. *Front. Psychol.* **9**, (2018). 10.3389/fpsyg.2018.01561 PMC6129743 30233451

[14] Tversky, A. & Kahneman, D. Judgment under uncertainty: Heuristics and biases. *Science* **185**, 1124–1131 (1974). 17835457 10.1126/science.185.4157.1124

[15] Samanez-Larkin, G. R. & Knutson, B. Decision making in the ageing brain: Thanges in affective and motivational circuits. *Nat. Rev. Neurosci.* **16**, 278–289 (2015). 25873038 10.1038/nrn3917 PMC5645075

[16] Tannou, T., Koeberlé, S., Aubry, R. & Haffen, E. How does decisional capacity evolve with normal cognitive aging: Systematic review of the literature. *Eur. Geriatr. Med.* **11**, 117–129 (2020). 32297227 10.1007/s41999-019-00251-8

[17] Nyberg, L., Lövdén, M., Riklund, K., Lindenberger, U. & Bäckman, L. Memory aging and brain maintenance. *Trends Cogn. Sci.* **16**, 292–305 (2012). 22542563 10.1016/j.tics.2012.04.005

[18] Roberts, K. L. & Allen, H. A. Perception and cognition in the ageing brain: A brief review of the short- and long-term links between perceptual and cognitive decline. *Front. Aging Neurosci.* **8**, 39 (2016). 26973514 10.3389/fnagi.2016.00039 PMC4772631

[19] Fjell, A. M., Sneve, M. H., Grydeland, H., Storsve, A. B. & Walhovd, K. B. The disconnected brain and executive function decline in aging. *Cereb. Cortex ***27**, 2303–2317 (2017). 27073220 10.1093/cercor/bhw082

[20] Depping, M. K. & Freund, A. M. Normal aging and decision making: The role of motivation. *Hum. Dev.* **54**, 349–367 (2011).

[21] Şi̇mşek, O., Kocak, O. & Younis, M. The impact of interpersonal cognitive distortions on satisfaction with life and the mediating role of loneliness. *Sustainability* **13**, 20 (2021).

[22] Leykin, Y., Roberts, C. S. & DeRubeis, R. J. Decision-making and depressive symptomatology. *Cogn. Ther. Res.* **35**, 333–341 (2011). 10.1007/s10608-010-9308-0 PMC3132433 21841849

[23] Lawlor, V. M. et al. Dissecting the impact of depression on decision-making. *Psychol. Med.* **50**, 1613–1622 (2020). 31280757 10.1017/S0033291719001570 PMC6946886

[24] Boyle, P. A. et al. Poor decision making is a consequence of cognitive decline among older persons without Alzheimer’s disease or mild cognitive impairment. *PloS One* **7**, e43647 (2012). 22916287 10.1371/journal.pone.0043647 PMC3423371

[25] Lee, D. Decision making: From neuroscience to psychiatry. *Neuron* **78**, 233–248 (2013). 23622061 10.1016/j.neuron.2013.04.008 PMC3670825

[26] Sun, T. et al. Decision-making under ambiguity or risk in individuals with Alzheimer’s disease and mild cognitive impairment. *Front. Psychiatry* **11**, (2020). 10.3389/fpsyt.2020.00218 PMC7093589 32256419

[27] Sutin, A. R., Aschwanden, D., Luchetti, M., Stephan, Y. & Terracciano, A. Sense of purpose in life and subjective cognitive failures. *Personal. Individ. Differ.* **200**, 111874 (2023). 10.1016/j.paid.2022.111874 PMC9988243 36891529

[28] Uğurlar, P., Sümer, N. & Posten, A. C. The cognitive cost of closeness: Interpersonal closeness reduces accuracy and slows down decision-making. *Eur. J. Soc. Psychol.* **51**, 1007–1018 (2021).

[29] Porcelli, A. J. & Delgado, M. R. Stress and decision making: Uffects on valuation, learning, and risk-taking. *Curr. Opin. Behav. Sci.* **14**, 33–39 (2017). 28044144 10.1016/j.cobeha.2016.11.015 PMC5201132

[30] Chi, W. C., Wolff, J., Greer, R. & Dy, S. Multimorbidity and decision-making preferences among older adults. *Ann. Fam. Med.* **15**, 546–551 (2017). 29133494 10.1370/afm.2106 PMC5683867

[31] Posadzy, K. *Social and Economic Factors in Decision Making under Uncertainty: Five Essays in Behavioral Economics* (Linköping University, Linköping, Sweden, 2017). 10.3384/diss.diva-143035.

[32] Davis-Stober, C. P., McCarty, K. N. & McCarthy, D. M. Decision making and alcohol: Health policy implications. *Policy Insights Behav. Brain Sci.* **6**, 64–71 (2019). 34295966 10.1177/2372732218818587 PMC8294170

[33] Mandolesi, L. et al. Effects of physical exercise on cognitive functioning and well-being: Biological and psychological benefits. *Front. Psychol.* **9**, (2018). 10.3389/fpsyg.2018.00509 PMC5934999 29755380

[34] Kasuga, H. et al. Association between participation in sports club activities and decision-making preferences in end-of-life treatment among Japanese elderly people: A cross-sectional study. *Fukushima J. Med. Sci.* **67**, 135–142 (2021). 34744089 10.5387/fms.2021-16 PMC8784194

[35] Cabeza, R. et al. Cognitive neuroscience of healthy aging: Maintenance, reserve, and compensation. *Nat. Rev. Neurosci.* **19**, 701–710 (2018). 30305711 10.1038/s41583-018-0068-2 PMC6472256

[36] Lövdén, M., Fratiglioni, L., Glymour, M. M., Lindenberger, U. & Tucker-Drob, E. M. Education and cognitive functioning across the life span. *Psychol. Sci. Public. Interest.* **21**, 6–41 (2020). 32772803 10.1177/1529100620920576 PMC7425377

[37] Boyle, P. A., Wilson, R. S., Yu, L., Buchman, A. S. & Bennett, D. A. Poor decision making is associated with an increased risk of mortality among community-dwelling older persons without dementia. *Neuroepidemiology* **40**, 247–252 (2013). 23364306 10.1159/000342781 PMC3760500

[38] Caudle, D. D. et al. Cognitive errors, symptom severity, and response to cognitive behavior therapy in older adults with generalized anxiety disorder. *Am. J. Geriatr. Psychiatry Off J. Am. Assoc. Geriatr. Psychiatry* **15**, 680–689 (2007). 10.1097/JGP.0b013e31803c550d 17670997

[39] Santangelo, G. et al. Subjective cognitive failures and their psychological correlates in a large Italian sample during quarantine/self-isolation for COVID-19. *Neurol. Sci.* **42**, 2625–2635 (2021). 33914195 10.1007/s10072-021-05268-1 PMC8082482

[40] Ruggeri, K. et al. The persistence of cognitive biases in financial decisions across economic groups. *Sci. Rep.* **13**, 10329 (2023). 37365245 10.1038/s41598-023-36339-2 PMC10293260

[41] Yates, J. F. & de Oliveira, S. Culture and decision making. *Organ. Behav. Hum. Decis. Process.* **136**, 106–118 (2016). 32288179 10.1016/j.obhdp.2016.05.003 PMC7126161

[42] Mohanty, S., Patnaik, B. C. M., Satpathy, I. & Sahoo, S. K. Cognitive biases and financial decisions of potential investors during Covid-19: An exploration. *Arab Gulf J. Sci. Res.* ahead-of-print (2023).

[43] Choudhary, S., Yadav, M. & Srivastava, A. P. Cognitive biases among millennial Indian investors: Do personality and demographic factors matter?. *FIIB Bus. Rev.* **13**, 106–117 (2024).

[44] Tairi, T. Associations between cognitive errors and mental health status in New Zealand adolescents. *Behav. Cogn. Psychother.* **48**, 280–290 (2020). 31718722 10.1017/S1352465819000626

[45] Komura, T., Cowden, R. G., Chen, R., Andrews, R. M. & Shiba, K. Estimating the heterogeneous effect of life satisfaction on cognitive functioning among older adults: Evidence of US and UK national surveys. *SSM - Ment. Health* **4**, 100260 (2023).

[46] International Institute for Population Sciences. Data User Guide - Longitudinal Ageing Study in India (LASI) Wave 1, 2017-18 (2020).

[47] Blanco, F. Cognitve Bias. In*Encyclopedia of Animal Cognition and Behavior*. 10.1007/978-3-319-47829-6 (2017).

[48] Jones, B. D. Bounded rationality. *Annu. Rev. Polit. Sci.* **2**, 297–321 (1999).

[49] Simon, H. A. Theories of Bounded Rationality. *Decision and Organization* 161–176 Preprint at (1972).

[50] Barros, G. & Herbert, A. Simon and the concept of rationality: Toundaries and procedures. *Braz. J. Polit. Econ.* **30**, 455–472 (2010).

[51] Kahneman, D., Slovic, P. & Tversky, A. *Judgment under Uncertainty: Heuristics and Biases* (Cambridge University Press(CUP), 1982). 10.1126/science.185.4157.1124 17835457

[52] Besedeŝ, T., Deck, C., Sarangi, S. & Shor, M. Age effects and heuristics in decision making. *Rev. Econ. Stat.* **94**, 580–595 (2012). 22544977 10.1162/REST_a_00174 PMC3337688

[53] Lo, A. W. & Zhang, R. The evolutionary origin of Bayesian heuristics and finite memory. *iScience* **24**, (2021). 10.1016/j.isci.2021.102853 PMC8340130 34381977

[54] Hjeij, M. & Vilks, A. A brief history of heuristics: How did research on heuristics evolve? *Humanit. Soc. Sci. Commun.* **10**, 1–15 (2023).

[55] Barash, J., Brocas, I., Carrillo, J. D. & Kodaverdian, N. Heuristic to bayesian: The evolution of reasoning from childhood to adulthood. *J. Econ. Behav. Organ.* **159** (2019).

[56] Lu, Y. The conjunction and disjunction fallacies: Explanations of the linda problem by the equate-to-differentiate model. *Integr. Psychol. Behav. Sci.* **50**, 507–531 (2016). 26077336 10.1007/s12124-015-9314-6 PMC4967104

[57] Muhammad, T. & Meher, T. Association of late-life depression with cognitive impairment: Evidence from a cross-sectional study among older adults in India. *BMC Geriatr.* **21**, 1–13 (2021). 34130632 10.1186/s12877-021-02314-7 PMC8204463

[58] Yaffe, K. et al. Depressive symptoms and cognitive decline in nondemented elderly women: A prospective study. *Arch. Gen. Psychiatry* **56**, 425–430 (1999). 10232297 10.1001/archpsyc.56.5.425

[59] Crane, M. K., Bogner, H. R., Brown, G. K. & Gallo, J. J. The link between depressive symptoms, negative cognitive bias and memory complaints in older adults. *Aging Ment. Health* **11**, 70715 (2007). 10.1080/13607860701368497 PMC2825049 18074258

[60] Sebind Kumar, A. N., Thoppil, S. P., Parassery, R. P. & Kunnukattil, S. S. Brief communication screening for depression among community–dwelling elders: Usefulness of the center for epidemiologic studies depression scale. *Indian J. Psychol. Med.* **38**, 483–485 (2016). 27833238 10.4103/0253-7176.191380 PMC5052968

[61] Zainal, N. H. & Newman, M. G. Life satisfaction prevents decline in working memory, spatial cognition, and processing speed: Latent change score analyses across 23 years. *Eur. Psychiatry* **65**, 1–13 (2022). 35437134 10.1192/j.eurpsy.2022.19 PMC9121850

[62] Zhu, X. et al. Satisfaction with life and risk of dementia: Findings from the Korean Longitudinal Study of Aging. *J. Gerontol. B Psychol. Sci. Soc. Sci.* **77**, 1831–1840 (2022). 35474537 10.1093/geronb/gbac064 PMC9535771

[63] Xiao, Y. et al. Cognition impairment prior to errors of working memory based on event-related potential. *Front. Behav. Neurosci.* **13**, (2019). 10.3389/fnbeh.2019.00013 PMC6379263 30809135

[64] Payne, T. W. & Schnapp, M. A. The relationship between negative affect and reported cognitive failures. *Depress. Res. Treat.*10.1155/2014/396195 (2014). 24669318 10.1155/2014/396195 PMC3942281

[65] International Institute for Population Sciences (IIPS), NPHCE, MoHFW,H. T. H. C. S. of P. H. (HSPH) and the U. of S. C. (USC). *Longitudinal Ageing Study in India (LASI) Wave 1, 2017-18, India Report*. (2020).

[66] Pandav, R., Fillenbaum, G., Ratcliff, G., Dodge, H. & Ganguli, M. Sensitivity and specificity of cognitive and functional screening instruments for dementia: The Indo-U.S. dementia epidemiology study. *J. Am. Geriatr. Soc.* **50**, 554–561 (2002). 11943056 10.1046/j.1532-5415.2002.50126.x

[67] Lefebvre, M. F. Cognitive distortion and cognitive errors in depressed psychiatric and low back pain patients. *J. Consult. Clin. Psychol.* **49**, 517–525 (1981). 6455451 10.1037//0022-006x.49.4.517

[68] Kim, D. Correlation between physical function, cognitive function, and health-related quality of life in elderly persons. *J. Phys. Ther. Sci.* **28**, 1844–1848 (2016). 27390430 10.1589/jpts.28.1844 PMC4932071

[69] Arokiasamy, P., Bloom, D., Lee, J., Feeney, K. & Ozolins, M. *Longitudinal Aging Study in India: Vision, Design, Implementation, and Preliminary Findings. Aging in Asia: Findings from New and Emerging Data Initiatives* (National Academies Press (US), 2012). 23077756

[70] Cervilla, J. A., Prince, M. & Mann, A. Smoking, drinking, and incident cognitive impairment: A cohort community based study included in the Gospel Oak project. *J. Neurol. Neurosurg. Psychiatry* **68**, 622–626 (2000). 10766894 10.1136/jnnp.68.5.622 PMC1736927

[71] Wu, J. et al. Relation of cigarette smoking and alcohol drinking in midlife with risk of cognitive impairment in late life: The Singapore Chinese Health Study. *Age Ageing* **48**, 101 (2019). 30307472 10.1093/ageing/afy166 PMC6322505

[72] Lin, Y. K., Peters, K. & Chen, I. H. Television watching, reading, cognition, depression and life satisfaction among middle-aged and older populations: A group-based trajectory modelling analysis of national data. *Health Soc. Care Community* **30**, e5661–e5672 (2022). 36057964 10.1111/hsc.13993

[73] Chang, Y. H., Wu, I. C. & Hsiung, C. A. Reading activity prevents long-term decline in cognitive function in older people: Evidence from a 14-year longitudinal study. *Int. Psychogeriatr.* **33**, 63–74. 10.1017/S1041610220000812 PMC8482376 32498728

[74] Blake, E., Dobson, K. S., Sheptycki, A. R. & Drapeau, M. The relationship between depression severity and cognitive errors. *Am. J. Psychother.* **70**, 203–221 (2016). 27329407 10.1176/appi.psychotherapy.2016.70.2.203

[75] Leung, P., Orgeta, V., Musa, A. & Orrell, M. Emotional distress mediates the relationship between cognitive failures, dysfunctional coping, and life satisfaction in older people living in sheltered housing: A structural equation modelling approach. *Int. J. Geriatr. Psychiatry* **34**, 179–185 (2019). 30259566 10.1002/gps.5007

[76] O’Rourke, N., Sixsmith, A., Kirshner, G. & Osher, Y. Perceived cognitive failures and quality of life for older adults with bipolar disorder. *J. Affect. Disord.* **287**, 433–440 (2021). 33862304 10.1016/j.jad.2021.03.024

[77] Culpepper, L., Lam, R. W. & McIntyre, R. S. Cognitive impairment in patients with depression: Awareness, assessment, and management. *J. Clin. Psychiatry* **78**, 3185 (2017). 10.4088/JCP.tk16043ah5c 29345866

[78] Crawford, T. J. et al. The effects of previous error and success in Alzheimer’s disease and mild cognitive impairment. *Sci. Rep.* **9**, 20204 (2019). 31882919 10.1038/s41598-019-56625-2 PMC6934582

[79] Delaney, R., Strough, J., Parker, A. M. & de Bruin, W. B. Variations in decision-making profiles by age and gender: A cluster-analytic approach. *Personal. Individ. Differ.* **85**, 19–24 (2015). 10.1016/j.paid.2015.04.034 PMC4438778 26005238

[80] Villanueva-Moya, L. & Expósito, F. Gender differences in decision-making: The effects of gender stereotype threat moderated by sensitivity to punishment and fear of negative evaluation. *J. Behav. Decis. Mak.* **34**, 706–717 (2021).

[81] Okamoto, S. et al. Decomposition of gender differences in cognitive functioning: National Survey of the Japanese elderly. *BMC Geriatr.* **21**, 38 (2021). 33423660 10.1186/s12877-020-01990-1 PMC7798327

[82] Bruch, E. & Feinberg, F. Decision-making processes in social contexts. *Annu. Rev. Sociol.* **43**, 207–227 (2017). 28785123 10.1146/annurev-soc-060116-053622 PMC5543983

[83] Wang, P. & Tan, C. The effects of social class on individuals’ decision-making tendencies in a prestige-money game: Social value or instrumental value?. *J. Gambl. Stud.* **35**, (2019). 10.1007/s10899-019-09827-2 30632011

[84] Lee, D. & Jackson, M. The simultaneous effects of socioeconomic disadvantage and child health on children’s cognitive development. *Demography* **54**, 1845–1871 (2017). 28836169 10.1007/s13524-017-0605-z PMC5856460

[85] Hagan, K. et al. Cumulative social disadvantage and health-related quality of life: National health interview survey 2013–2017. *BMC Public. Health* **23**, 1710 (2023). 37667245 10.1186/s12889-023-16168-8 PMC10476290

[86] Röhr, S. et al. Social determinants and lifestyle factors for brain health: Implications for risk reduction of cognitive decline and dementia. *Sci. Rep.* **12**, 12965 (2022). 35902604 10.1038/s41598-022-16771-6 PMC9334303

[87] Liu, H., Zhang, Y., Burgard, S. A. & Needham, B. L. Marital status and cognitive impairment in the United States: Evidence from the National Health and Aging trends Study. *Ann. Epidemiol.* **38**, 28–34e2 (2019). 31591027 10.1016/j.annepidem.2019.08.007 PMC6812624

[88] Jennings, E. A., Farrell, M. T., Liu, Y. & Montana, L. Associations between cognitive function and marital status in the United States, South Africa, Mexico, and China. *SSM - Popul. Health* **20**, 101288 (2022). 36444339 10.1016/j.ssmph.2022.101288 PMC9700314

[89] Saenz, J. L., Downer, B., Garcia, M. A. & Wong, R. Rural/urban dwelling across the life-course and late-life cognitive ability in Mexico. *SSM - Popul. Health* **17**, 101031 (2022). 35118187 10.1016/j.ssmph.2022.101031 PMC8800130

[90] Jin, M., Ji, L. & Peng, H. The relationship between cognitive abilities and the decision-making process: The moderating role of self-relevance. *Front. Psychol.* **10**, 1892 (2019). 31474917 10.3389/fpsyg.2019.01892 PMC6702323

[91] Harling, G. et al. Social contact, social support, and cognitive health in a population-based study of middle-aged and older men and women in rural South Africa. *Soc. Sci. Med.* **260**, 113167 (2020). 32688161 10.1016/j.socscimed.2020.113167 PMC7441312

[92] Hikichi, H., Kondo, K., Takeda, T. & Kawachi, I. Social interaction and cognitive decline: Results of a 7-year community intervention. *Alzheimers Dement. Transl. Res. Clin. Interv.* **3**, 23–32 (2017). 10.1016/j.trci.2016.11.003 PMC5651375 29067317

[93] Smith, T. W., Follick, M. J., Ahern, D. K. & Adams, A. Cognitive distortion and disability in chronic low back pain. *Cogn. Ther. Res.* **10**, 201–210 (1986).

[94] Zheng, J., Liu, J. & An, R. Functional limitation and cognitive impairment among 80 + year old Chinese. *Australas. J. Ageing* **35**, 266–272 (2016). 27320413 10.1111/ajag.12341

[95] Gildner, T. E., Liebert, M. A., Kowal, P., Chatterji, S. & Snodgrass, J. J. Associations between sleep duration, sleep quality, and cognitive test performance among older adults from six middle income countries: Results from the study on Global Ageing and Adult Health (SAGE). *J. Clin. Sleep. Med.* **10**, 613–621 (2014). 24932140 10.5664/jcsm.3782 PMC4031401

[96] Kumar, M., Srivastava, S. & Muhammad, T. Relationship between physical activity and cognitive functioning among older Indian adults. *Sci. Rep.* **12**, 2725 (2022). 35177736 10.1038/s41598-022-06725-3 PMC8854730

[97] Law, C. K., Lam, F. M., Chung, R. C. & Pang, M. Y. Physical exercise attenuates cognitive decline and reduces behavioural problems in people with mild cognitive impairment and dementia: A systematic review. *J. Physiother.* **66**, 9–18 (2020). 31843427 10.1016/j.jphys.2019.11.014

[98] Benito-León, J., Ghosh, R., Lapeña-Motilva, J., Martín-Arriscado, C. & Bermejo-Pareja, F. Association between cumulative smoking exposure and cognitive decline in non-demented older adults: NEDICES study. *Sci. Rep.* **13**, 5754 (2023). 37031269 10.1038/s41598-023-32663-9 PMC10082795

[99] Nadar, M., Sh., Hasan, A. M. & Alsaleh, M. The negative impact of chronic tobacco smoking on adult neuropsychological function: A cross-sectional study. *BMC Public Health* **21**, 1278 (2021). 34193083 10.1186/s12889-021-11287-6 PMC8247072

[100] Rodriguez-Sickert, C. Homo Economicus. In *Handbook of Economics and Ethics* (Edward Elgar Publishing, 2009).

