# Author Correction: Late-life psychiatric factors and life satisfaction are associated with cognitive errors: evidence from an experimental module of a large-scale survey in India

**DOI:** 10.1038/s41598-024-78825-1

**Published:** 2024-11-15

**Authors:** C. V. Irshad, P. Padma Sri Lekha, E. P. Abdul Azeez, T. Muhammed

**Affiliations:** 1 School of Social Sciences and Languages, Vellore Institute of Technology, Vellore, Tamil Nadu 632014 India; 2 Department of Human Development and Family Studies, Pennsylvania State University, University Park, PA 16802 USA

Correction to: *Scientific Reports* 10.1038/s41598-024-76180-9, published online 29 October 2024

The original version of this Article contained an error in the Abstract.

“Depression (aOR = 1.28, 99%, CI: 1.16–1.41), life satisfaction (aOR = 0.99, 99%, CI: 0.98–1.00), and cognitive impairment (aOR = 1.13, 90% CI: 1.00–1.30) self-reported psychiatric) were significantly associated with higher odds of committing cognitive errors among the middle-aged and older adults.”

now reads:

“Depression (aOR = 1.28, 99%, CI: 1.16–1.41), life satisfaction (aOR = 0.99, 99%, CI: 0.98–1.00), and cognitive impairment (aOR = 1.13, 90% CI: 1.00–1.30) were significantly associated with higher odds of committing cognitive errors among the middle-aged and older adults.”

The original Article has been corrected.

